# Photobleaching of Imidazole Brown Carbon in Single Levitated Aerosol Particles

**DOI:** 10.1021/acs.jpca.6c01327

**Published:** 2026-06-01

**Authors:** Xu Zhang, Padraig E. Meehan, Jamie W. Knight, Andrew J. Orr-Ewing, Michael I. Cotterell

**Affiliations:** † School of Chemistry, 1980 University of Bristol, Bristol BS8 1TS, U.K.; ‡ College for Engineering, Mathematics and Physical Sciences, University of Exeter, Exeter EX4 4QF, U.K.; § Department of Chemistry, University of Oxford, Oxford OX1 3QZ, U.K.

## Abstract

Brown carbon (BrC) aerosols are key contributors to atmospheric light absorption and photochemistry, yet their optical properties and photochemical behavior are poorly understood. To explore the influence of a microdroplet environment on the photochemical decay of a representative BrC species, single particle cavity ring-down spectroscopy was used to probe individual aerosol particles containing imidazole-2-carboxaldehyde (IC) confined inside a linear electrodynamic quadrupole balance. The aerosol particles were levitated and exposed to 405 nm wavelength laser light to drive photobleaching. The imaginary component of the complex refractive index (*k*) provided a direct characterization of the aerosol particle light absorption and was determined for varying exposures to the photolyzing light. The determined *k* values decayed exponentially with exposure time, demonstrating photobleaching. A kinetic model incorporating Lorenz–Mie theory was fitted to the observed decay to obtain a photobleaching quantum yield (within the framework of the kinetic model) for IC of (9.6 ± 3.0) × 10^–5^. The developed kinetic framework can be used to predict photobleaching time scales in aerosols from knowledge of these effective quantum yields. Additional photobleaching measurements on bulk IC-containing solutions showed that the photobleaching quantum yields are an order of magnitude larger in aerosol droplets, potential reasons for which are discussed.

## Graphical abstract

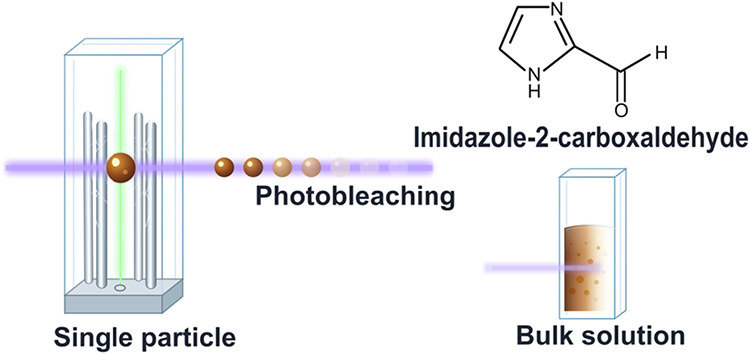

## 1 Introduction

Atmospheric aerosols play a critical role in modulating Earth’s energy balance, and hence its climate, as well as impacting air quality. They affect radiative forcing by scattering and absorbing solar radiation directly and serving as cloud condensation nuclei. Despite their recognized significance, aerosols remain one of the largest sources of uncertainty in global climate models, particularly because of the difficulty of quantifying their net radiative forcing.^1^ The scattering of sunlight by aerosol (with a corresponding net negative radiative forcing) compensates partly for the positive radiative forcing of greenhouse gases, but uncertainty in the magnitude of aerosol radiative forcing prevents more accurate predictions of future atmospheric temperatures.^2^ Among the wide variety of aerosol species, light-absorbing organic aerosols known as brown carbon (BrC) represent a particularly poorly constrained component. BrC aerosols are comprised of a diverse range of light absorbing organic species that exhibit characteristic strong absorption at short visible wavelengths, compared to weak absorption at longer visible wavelengths, giving them a brown color. Estimates of BrC radiative forcings vary by a factor of ∼15 (from 0.04 to 0.57 W m^–2^).^3,4^ This large variation derives from uncertainties in the optical properties of BrC and how they evolve over aerosol particle lifetime.^3^ The primary cause of this uncertainty in optical properties lies in the diversity of BrC species, with BrC describing a broad category of chromophoric organic molecules originating from multiple sources, including primary emissions from biomass burning and fossil fuel combustion, as well as secondary organic aerosol formation. Biomass burning alone accounts for approximately 74% of global organic carbon aerosol emissions.^5^ In addition to climate impacts, BrC plays an important role in atmospheric chemistry. Absorption of UV and short-wavelength visible sunlight by BrC can interfere with photolysis rates of key atmospheric oxidants such as ozone and nitrogen dioxide, altering radical budgets and thereby affecting air quality and secondary pollutant formation.^6^ Understanding the composition and reactivity of BrC is therefore not only essential for improving climate models, but also for evaluating its broader implications for atmospheric oxidation capacity and human health.

Imines are a class of BrC chromophores that have attracted increasing attention in recent years. Imine species contain the functional group R_2_C=N-R (such as imidazole) and are proposed to form in the aqueous phase through the reaction of nitrogen-containing species (such as NH_3_, (NH_4_)_2_SO_4_, and amines) with carbonyl compounds, particularly glyoxal and methylglyoxal.^7–11^ These reactions, though thermodynamically favorable, proceed slowly under typical bulk aqueous conditions, with reaction time scales on the order of weeks and requiring high solute concentrations (∼1 M).^8,12^ However, imine formation rates in aerosol droplets may exceed those observed in macroscopic bulk solutions. This discrepancy likely results from aerosols accessing metastable supersaturated concentrations of dissolved reactants during humidity cycling (e.g., cloud processing).^7,13^ Additionally, laboratory studies have shown that glyoxal uptake onto ammonium sulfate particles can proceed on time scales of minutes, suggesting efficient surface-mediated formation of imidazoles in real atmospheric aerosols;^14–17^ this formation pathway becomes important at the high surface-to-volume-ratios of atmospheric aerosol particles.

Imidazole and its derivatives – particularly imidazole-2-carboxaldehyde (IC) – have been identified in ambient aerosol samples at concentrations up to ∼14 ng m^–3^.^18^ These species are of special interest not only due to their absorptivity but also their potential photochemical reactivity. In particular, IC is a known photosensitizer, undergoing excitation to a reactive triplet state. Time-resolved spectroscopy and flash photolysis studies indicate triplet-state lifetimes of IC in the microsecond range.^19,20^ The triplet-state molecules can participate in hydrogen atom transfer reactions and radical formation, and can promote aqueous phase oxidation of dissolved sulfur species to H_2_SO_4_.^21–25^ Moreover, the reactive triplet state may initiate secondary photochemical processes in the condensed phase such as in aerosol particles.^19,25^

Despite the atmospheric relevance of imidazole photochemistry, most experimental studies have been conducted in bulk solutions or using filter-extracted surrogates.^26–28^ Zhao et al. prepared aqueous bulk solutions containing imine chromophores produced from the reaction of ammonium sulfate with glyoxal or methylglyoxal.^26^ The observed decay times for both photolysis and hydroxyl oxidation processes ranged from several minutes to several hours. Measurements by Aiona et al. on similar reaction products found the imine samples were photobleached rapidly, with the authors estimating the equivalent photolysis lifetimes of the imines as ∼13 min at zero solar zenith angle at sea level.^27^ Wong et al. collected imine BrC aerosol particles generated from bulk aqueous phase solution reactions of ammonium sulfate with methylglyoxal on filters and extracted the organic components of these particles into solution.^28^ Using chromatography coupled with ultraviolet–visible spectroscopy, the authors characterized the variation in UV/vis absorption spectra with irradiation time following molecular weight separation. The imine brown carbon chromophores were photobleached with a corresponding half-life of 95 min.

While studies on bulk solutions offer molecular insights, they exhibit significant limitations when scaling photochemical behaviors to the unique aerosol environment. Aerosol particles commonly exist in states, and exhibit properties, that are inaccessible to bulk samples and yet affect their aging. Aerosol particles are mesoscopic compartments, the behaviors of which are often dominated by surface processes; aerosol diameters range from ∼10 nm to 100 μm with surface area to volume ratios up to 10^7^ times greater than at the macroscopic condensed-phase scale (∼1 L).^29^ High surface-to-volume ratios lead to the preferential partitioning of surface-active species to the particle-gas interface in liquid droplets. Enrichment and molecular ordering of reactants at these surfaces have been linked to faster reactions compared to those observed in bulk solutions.^30^ Liquid droplets lack rough surfaces to nucleate crystal formation, allowing metastable supersaturated concentrations of solutes to be accessed on removal of solvent, with solute concentrations that can be >10^3^ higher than those achieved in macroscopic solutions.^29^ In addition to the impacts that supersaturation of reacting solutes could have on reaction rates, altered rheology and inhibited molecular diffusion are commonplace in these solutions through the formation of kinetically arrested glassy states that are inaccessible in macroscopic samples.^31^ Furthermore, aerosol droplets exhibit unique optical phenomena; light incident on spherical droplets can excite internal standing wave modes, forming complex electromagnetic field distributions (referred to as morphology dependent resonances) that affect photoinitiated in-droplet chemistry.^32^ Therefore, studies of BrC aging should prioritize direct investigation of BrC-containing aerosol particles to capture accurately the relevant time scales and mechanisms of photobleaching.

Spectroscopic interrogation of single levitated particles provides direct and *in situ* measurements of aerosol properties in tightly regulated environments and opens new avenues for unravelling the effects of the unique aerosol molecular environment on photochemical reactivity. Single-particle spectroscopy has emerged as a powerful approach for probing light-induced transformations, including photobleaching and photodegradation. Using photoacoustic spectroscopy, Cremer and co-workers demonstrated that nanofocusing of light within optically trapped dye-containing particles can accelerate photobleaching processes.^32–34^ Other single-particle studies revealed similar size-dependent enhancements in photochemical activity, such as the photoreduction of iron­(III)–citrate aerosols driven by internal optical field effects,^35^ and the photodegradation of carminic acid within aerosol particles examined via photophoretic spectroscopy.^36^ Moreover, light-scattering and Raman spectroscopic investigations have shown that even nominally nonabsorbing droplets, such as those composed of oleic acid or amino acid mixtures, can undergo significant photochemical transformation under visible irradiation.^37–40^ However, to the best of our knowledge, no single aerosol particle study has sought to interrogate the photolytic aging of BrC species and their evolving optical properties.

This work investigates the photochemistry of a representative brown carbon species contained inside an aerosol particle, focusing on the behavior of IC. Our previous studies successfully used single particle cavity ring-down spectroscopy (SP-CRDS) to measure directly the evolving extinction cross sections of light absorbing particles levitated inside a linear electrodynamic quadrupole (LEQ) balance, as their size and chromophore concentrations changed.^41–44^ Here, we report the use of this SP-CRDS-LEQ approach to examine the photobleaching kinetics of IC in single aerosol droplets irradiated by 405 nm wavelength photolyzing radiation. Section S4 in the Supporting Information shows that light absorption by IC extends, albeit weakly, into this blue region of the visible spectrum. Moreover, we describe a model framework to predict the evolving aerosol absorption and determine the photobleaching quantum yields of IC in the unique physicochemical environments of aerosols.

## 2 Experimental Methods

To determine the photobleaching rate of IC in single aerosol droplets, we quantified the evolving imaginary component of the complex refractive index as a function of exposure time to photolyzing radiation, which served as a direct indicator of changes in droplet chemical composition. Particles composed only of IC are solid and nonspherical, which presents challenges to the interpretation of scattering measurements. Instead, we doped spherical droplets of 1,2,6-hexanetriol (HT; a semivolatile, liquid organic species) with small concentrations of IC; the HT and IC are miscible, and form homogeneously mixed aerosol droplets. The real and imaginary components of the refractive index for these aerosol droplets were obtained by accurately and precisely measuring particle size-dependent extinction cross sections using SP-CRDS and comparing the results with predictions from Lorenz-Mie theory. Section 2.1 provides an overview of the SP-CRDS instrument setup. Section 2.2 outlines the procedure for retrieving the complex refractive index of single droplets via a grid search algorithm, with special consideration given to the retrieval of imaginary refractive indices for weakly absorbing particles. Section 2.3 describes the method used to acquire UV/vis absorption spectra of IC-HT bulk solutions, from which the wavelength-dependent imaginary component of refractive index of IC was determined.

### 2.1 Single Particle Cavity Ring-Down Spectroscopy

Our bespoke instrument enabled the optical properties of single light-absorbing aerosol droplets to be interrogated over indefinite time scales. This instrument and associated data processing techniques have been presented in our previous publications.^41–43^ Our approach used a LEQ balance to levitate a single aerosol droplet (with chosen initial radii in the range 1.3–1.5 μm) at the center of a 405 nm wavelength probe beam (Toptica Photonics, TopMode 50 mW) inside a high-finesse optical cavity, as shown in Figure 1. CRDS provided direct, calibration-free measurements of the particle extinction cross-section (σ_ext_). Bulk stock solutions containing both IC (Thermo Scientific, CAS code: 10111-08-7, 97%) and HT (Sigma-Aldrich, CAS code: 196-69-4, 96%) were made with HPLC-plus water (Sigma-Aldrich, CAS code: 7732-18-5). The total solute mass concentration of these solutions was 0.05% (w/w), and the mass ratio of IC and HT was 6:10. A droplet-on-demand dispenser generated aqueous droplets with highly reproducible initial diameters of 20–25 μm. A DC voltage applied to an induction ring-electrode positioned 2–5 mm from the dispenser tip induced a charge of several femtocoulombs on the droplets. This charge enabled the manipulation of the droplet position using electric fields exerted by electrodes within the LEQ trapping cell. The charged aqueous droplets were injected into the center of the LEQ trap, where their radii decreased rapidly to ∼1.5 μm by evaporation of their water content in the low relative humidity (RH) environment maintained at <10% by purging the sample volume with N_2_. Subsequent size changes were dominated by the slow evaporation of the HT and IC from the droplet.

**Figure 1:**
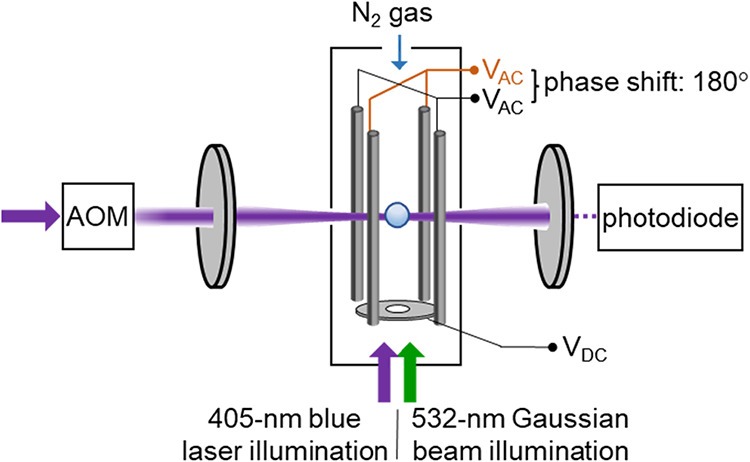
Schematic diagram of the single particle CRDS instrument. AOM is an acousto-optic modulator. *V*_AC_ and *V*_DC_ denote the application of alternating current and direct current voltages, respectively, to electrodes.

The trapping cell consisted of four vertical rods arranged in a square array, and a ring electrode positioned at the base of the cell (see Figure 1). By applying high-frequency alternating current voltages to pairs of diametrically opposed rods, with a 180-degree phase shift in the sinusoidal voltage waveform between each pair, the charged droplets entering the cell were confined to a narrow cylindrical space centered on the LEQ symmetry axis.^43^ Repulsion by an adjustable DC voltage applied to the ring-electrode balanced the gravitational force of the droplets and a downward Stokes drag force exerted by the laminar flow of nitrogen gas, the RH of which was controlled. By adjusting the voltage applied to this ring electrode, the height of a chosen particle was controlled to position it at the center of our 405 nm CRDS spectrometer, thereby enabling single-particle extinction cross-section measurements. The position of the particle on the central axis of the LEQ was monitored by an imaging camera, and its height was maintained by a feedback loop that adjusted the DC voltage applied to the ring electrode in response to changes in droplet weight caused by slow particle evaporation.

A 532 nm cw laser beam with a power of 20 mW propagated vertically upward along the center axis of the LEQ trap, illuminating the suspended particle. The 532 nm wavelength was selected because IC exhibits negligible absorption at this wavelength, thereby minimizing direct photothermal or photochemical perturbations during the measurements. A camera coupled to a long working distance microscope objective with a numerical aperture of 0.42 recorded images of the angularly resolved *s*-polarized elastically scattered light intensity over an angular range of approximately 65.2 to 114.8 degrees at a sampling rate of ∼10 Hz. These images were converted in real time to one-dimensional distributions of relative intensities with scattering angle, i.e., a phase function, from which the particle size was determined. An additional 405 nm wavelength laser beam (Toptica Photonics, iBeam smart 405-S 200 mW) was aligned to be colinear with the 532 nm laser, but with orthogonal polarization. This 405 nm laser drove the photochemical reactions of IC in trapped droplets. To prevent collection of 405 nm light by the phase function imaging camera, a linear polarizer (Thorlabs, NE04A) was introduced before the camera for collection of the *s*-polarized 532 nm light only.

The high-finesse linear optical cavity of the CRDS instrument was formed by two high-reflectivity (*R* > 99.99%) concave mirrors. 405 nm laser light from the TopMode laser was passed through an acousto-optic modulator (AOM) and the first-order diffraction beam was coupled into the cavity. The rear cavity mirror was mounted on a piezo-electric tube that constantly scanned the cavity length back and forth over distances of a few micrometres to drive the cavity periodically into resonance with the 405 nm excitation laser light. A small amount of light transmitted through the rear cavity mirror on each reflection was monitored with a photodiode. On excitation of a cavity mode, an electronic pulse was sent to the AOM to rapidly extinguish light intensity in the first-order diffraction beam and the intracavity light intensity then decayed exponentially with a characteristic ring-down time. The ring-down times for both the empty cavity (τ_0_) and for the particle levitated at the center of the cavity fundamental (TEM_00_) mode (τ) were recorded.

### 2.2 Retrieval of the Complex Refractive Index

The intensive optical properties of an aerosol particle are characterized by its effective complex refractive index, which comprises both real (*n*) and imaginary (*k*) components. The latter quantifies the light absorption by a material. Our SP-CRDS method retrieved values for both components of the complex refractive index from particle size-dependent extinction cross-section measurements. The ring-down times measured at a sampling rate of ∼30 Hz by CRDS were binned in 1-s intervals, and the mean ring-down time at a 1 Hz sampling rate was calculated from these binned data. These averaged ring-down time data were used to calculate the particle extinction cross sections using

1 $$\sigma_{\mathrm{ext}}=\frac{L\pi {w_{\mathrm{CRD}}}^{2}}{2c}\left(\frac{1}{\tau }-\frac{1}{\tau_{0}}\right)$$

in which *L* is the length of the optical cavity, *c* is the speed of light, and *w*_CRD_ is the focal beam waist for the TEM_00_ mode. Subsequently, the measured extinction cross section values were compared to predictions from Lorenz-Mie theory using a least-squares grid search approach. This grid search included variation of the real component of the complex refractive index and the value of *w*_CRD_ used to convert the ring-down times to extinction cross sections, as well as a small additive correction factor *r*_m_ to the phase function retrieved radii. The addition of *r*_m_ provides a small correction to account for any systematic biases in the phase function retrieved values. The imaginary component of the complex refractive index was set to an initial estimate obtained from a coarse four-parameter grid search.

The comparisons between the measured and modeled cross sections were evaluated using the merit function

2 $$\chi =\frac{1}{N}\sum_{i=1}^{N}{\left(\sigma_{\exp,i}-\sigma_{\mathrm{Mie},i}\right)}^{2}$$

with *N* the total number of data points, σ_exp,*i*_ the measured extinction cross-section at a given particle radius, and σ_Mie,*i*_ the Lorenz-Mie predicted cross-section at the same particle radius for a given complex refractive index. Both IC and HT are semivolatile molecules that evaporated steadily and continuously from droplets over time. During droplet evaporation, both the real and imaginary parts of the complex refractive index were assumed to remain constant (i.e., the concentrations of IC and HT in the droplets did not vary). As shown in Section 3, we find no evidence to suggest these parameters changed by detectable amounts over the course of an experiment after the initial period of exposure to the 405 nm photolyzing laser. The input parameters *n*, *w*_CRD_, and *r*_m_ collectively determined the optimal fitting values corresponding to the minimum value of χ.

After fully optimizing *n*, *w*_CRD_ and *r*_m_, the imaginary refractive index component *k* was independently refined using a merit function that quantifies the discrepancy between the Lorenz–Mie–predicted and experimentally observed peak heights of the Mie resonances in σ_ext_

3 $$\chi \left(k\right)=\frac{1}{N_{p}}\sum_{p=1}^{N_{p}}{\left(\sigma_{\mathrm{Mie}}\left(r_{p}\right)-\max_{r_{p}\;-△r/2\leq r\leq r_{p}\;+△r/2}\sigma_{\exp}\left(r\right)\right)}^{2}$$

in which *N*_p_ is the total number of synthetic resonance peaks considered, Δ*r* is a finite radius window (set to 4 nm) centered at *r*_p_ (the resonance peak position that was identified from the synthetic spectrum), accounting for potential peak shifts and finite experimental resolution. The optimal value of *k* was obtained by minimizing χ­(*k*) over the explored parameter range. This peak-based approach to retrieving *k* is resilient to small ∼ nanometre errors in particle radii retrieved from phase functions for the weakly absorbing droplets interrogated in this work, which exhibited *k* < 0.0015.

### 2.3 UV/vis Measurements on Bulk Solutions

Bulk Solutions of IC were prepared in HPLC-plus water (Sigma-Aldrich) or in 1-butanol (Acros Organics, 99.5%) to measure the absorbance of IC in aqueous and organic solvent environments, respectively. We note that the high viscosity of 1,2,6-hexanetriol precluded the preparation of a homogeneous solution of IC and HT for bulk UV/vis spectroscopy. Hence, we instead chose 1-butanol as a related organic, saturated-alcohol matrix. The concentrations of IC in the two solutions were 0.2 mM. The absorption spectra of the solutions were measured over the 200–800 nm wavelength range using an Agilent Cary 60 UV/vis spectrophotometer. All measurements were conducted at an ambient temperature of 20 ± 1 °C. A 1 cm path length Hellma quartz cuvette was employed, with the background spectrum acquired using the same cuvette filled with solvent.

## 3 Results and Discussion

In this section, we demonstrate photobleaching in single aerosol droplets composed of a binary mixture of IC and HT. Section 3.1 presents imaginary refractive index determinations for IC in bulk solution, and CRDS measurements for IC-HT aerosol droplets exposed to photolyzing radiation for varying durations. By maintaining a constant intensity of the photolyzing laser beam illuminating the droplets, we establish trends in *k* with exposure time that demonstrate photobleaching of the IC chromophore. Section 3.2 develops a kinetic model to describe the evolving *k*, which we fit to our data to reveal the photobleaching quantum yield for IC in our droplets. This model requires accurate characterizations of the intensity profile at the beam waist of the photolyzing laser beam. Therefore, we introduce a novel method for determining the intensity profile of this laser beam at the position of the trapped particle. We also report measurements of the photobleaching quantum yield of IC in bulk aqueous solution that differ from those of the aerosol measurements, the potential reasons for which are discussed.

### 3.1 Optical Measurements for Photobleached IC Aerosols

Figure 2a shows the absorption spectra of IC in water (blue line) and 1-butanol (red dashed line) across the 200–800 nm wavelength range, while Figure 2b shows the corresponding wavelength-dependent imaginary component of the refractive index for IC (*k*_IC_) calculated using the following procedure. The measured absorbance (*A*) is related to the imaginary component of the refractive index for the solution by

4 $$k=\frac{A\lambda \ln10}{4\pi l}$$

in which λ is the wavelength of light, and *l* is the optical path length (1 cm).^45^ By applying the mass fraction mixing rule to the imaginary part of the complex refractive index of the two-component (IC and solvent) solution, the *k* value of the IC solute is obtained using

5 $$k_{\mathrm{IC}}=\frac{k}{w_{\mathrm{IC}}}$$

in which *w*_IC_ is the mass fraction of IC in the bulk solution. This mass fraction mixing model assumes that the absorbances of either solvent can be ignored; this assumption is reasonable because both solvents are nonabsorbing at visible wavelengths of light and the background spectra, which were recorded and subtracted from the sample absorption spectra, used the same Hellma quartz cuvette filled with the corresponding solvent only.

**Figure 2:**
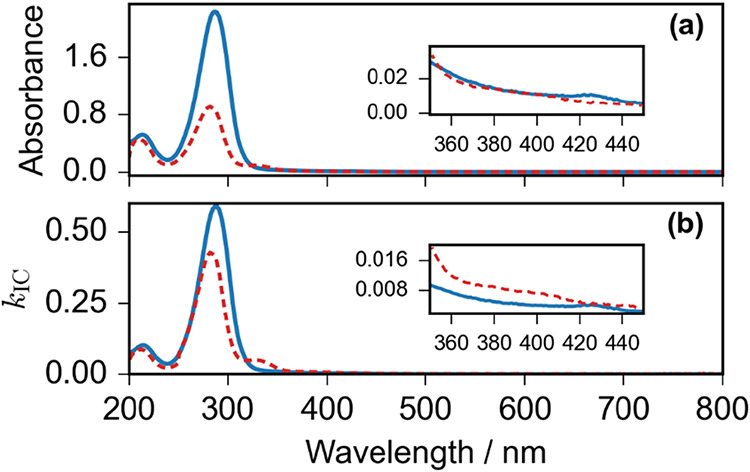
(a) Absorption spectra for IC solutions in water (0.2 mM, blue line) and 1-butanol (0.2 mM, red dashed line) measured using UV/vis absorption spectroscopy. The inset shows an expanded portion of the absorbance data over the wavelength range 350–450 nm. (b) Calculated imaginary component of the refractive index for IC using the method described in the main text.

The analysis described above must take into account the propensity for carbonyl compounds to form hemiacetals in alcohol solutions, or diols in aqueous solution, removing the carbonyl chromophore responsible for the long-wavelength n → π* absorption band, which extends beyond 400 nm in an aqueous solution of IC.^46^ As shown by Crespi et al.,^47^ IC does not form a diol in aqueous (D_2_O) solution, but 45% of IC molecules convert to the hemiacetal in CD_3_OD. In our analysis of the 1-butanol spectrum, the calculation of *k*_IC_ values for the weak n → π* absorption band and the π → π* absorption band peaking at 282 nm, which is also associated with the carbonyl chromophore, were corrected for hemiacetal formation by assuming the same 45% conversion (on a molar basis) as reported for IC in CD_3_OD. However, this correction was not applied to the short UV-wavelength absorption band evident below 250 nm because this feature is associated with π → π* excitation of the imidazole ring, which is present in both the keto and hemiacetal forms of IC.

The values of *k*_IC_ calculated using this approach at the 405 nm wavelength of interest here are 0.0039 and 0.0067 from measurements made in water and 1-butanol solution, respectively. The differences in *k*_IC_ at 405 nm are likely a consequence of the different solvent environments in water and 1-butanol, which affect the energies of the n → π* and π → π* transitions to different extents. The *k*_IC_ value in 1-butanol is particularly relevant to the aerosol droplet studies presented in the next section, which probe IC within an organic saturated alcohol (1,2,6-hexanetriol) matrix.

Figure 3 compares the particle size-dependent extinction cross sections for unbleached and bleached IC-HT aerosol droplets under a controlled RH of ∼10%. Temporal changes in droplet size were driven by the evaporation of IC and HT. Over the course of the ∼2-h measurement period, the particle radius decreased from approximately 1500 to 1100 nm due to continuous evaporation. The unbleached droplet was measured in the absence of any photolyzing light from the 405 nm photoexcitation laser. Meanwhile, for the bleached droplet, the 405 nm photoexcitation laser continuously illuminated the droplet over the total duration of the experiment. The assignment of absorption of this 405 nm light to IC in the droplets (albeit in the long-wavelength tail of the IC absorption spectrum), instead of to any trace impurities that might be present in our samples, is discussed in detail in the Supporting Information (SI Section S4). Data collection began 10 min after laser exposure; as we show below, photobleaching is completed after approximately 300 s for our droplets and illumination conditions and therefore the measurements in Figure 3b correspond to a droplet for which photobleaching was finished.

**Figure 3:**
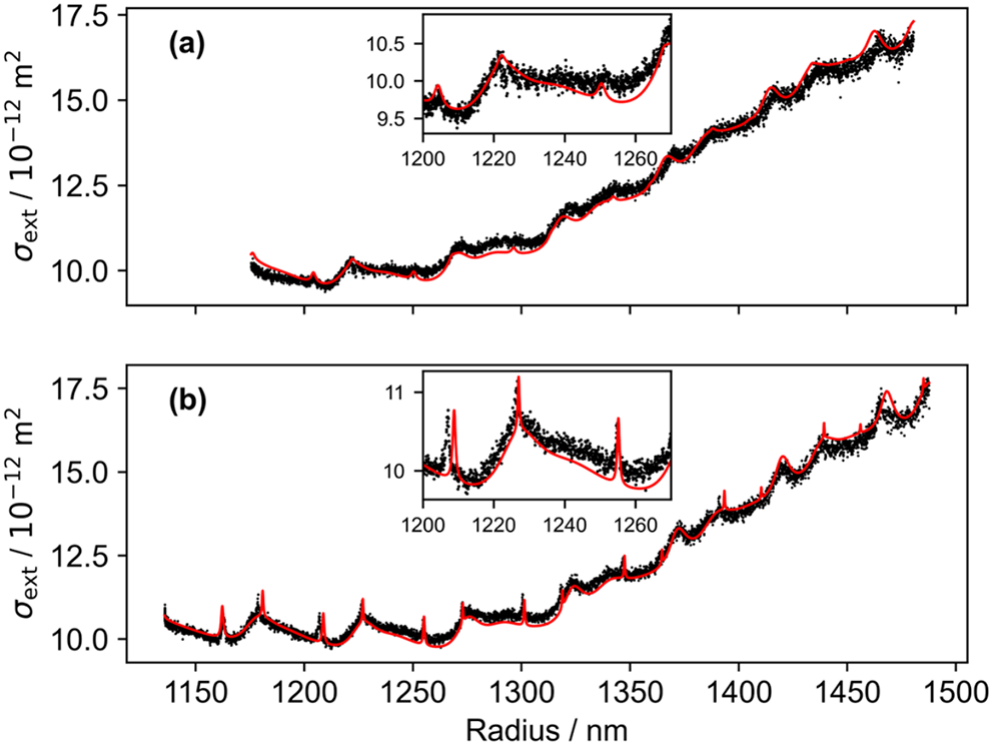
Comparison of measured (black points) particle size-dependent variations in extinction cross sections of (a) an unbleached, and (b) a fully bleached droplet of IC-HT under dry (∼10% RH) conditions. The red curves are fits to Lorenz-Mie theory, as described in Section 2.2 of the text.

Figure 3 also shows the best-fit predictions obtained from Lorenz-Mie theory. Both the real and imaginary components of the complex refractive index were assumed to remain constant throughout each individual measurement. In the bleached droplet data set in Figure 3b, the size-dependent extinction cross-section profiles exhibit broad oscillatory features arising from the interference between light passing around the droplet and light refracted through the droplet. Superimposed on the interference structure are sharp resonance structures, referred to as *ripple structure* or *Mie resonances*, resulting from efficient coupling of light into resonant optical modes of the spherical particles.^45^ These resonant and interference features are attenuated in the unbleached droplet data set, because absorption by IC reduces the quality factor of the droplet as an optical resonator and broadens and dampens the resonance features in the extinction and ripple structures. The retrieved *k* values for the unbleached and bleached particles were 0.00141 and 0.00018, respectively, demonstrating a significant reduction in *k* at the 405 nm wavelength of the CRDS probe and hence substantial photobleaching of IC under sustained 405 nm illumination.

To quantify the photobleaching rate, we determined how the value of *k* evolved for replicate measurements on individual, but different, droplets following fixed illumination periods of up to 360 s with the photolyzing 405 nm laser beam. To ensure precise control over the illumination time, each experiment was preceded by realignment of the photolyzing laser beam using a test droplet. The alignment of the photolyzing laser beam was optimized to maximize the 405 nm light intensity scattered from the test droplet, as measured by the phase function imaging camera. These alignment measurements were made in the absence of the 532 nm phase function imaging laser beam, and with the linear polarizer affixed to the camera imaging stage rotated to enable collection of the *p*-polarized 405 nm scattered light. The test droplet was then removed from the trap, the 532 nm phase function imaging laser beam reintroduced, the camera imaging polarizer rotated 90° for optimal collection of the *s*-polarized 532 nm light, and a second identical droplet was introduced into the trapping cell at the same position as the test droplet. This droplet was irradiated by the 405 nm photolyzing laser to initiate photochemical reactions for a fixed period. The estimated uncertainty in the recorded exposure time to the 405 nm light source was one second. The intensity of the photolyzing laser beam immediately before the LEQ trapping cell was measured using a calibrated power meter (Thorlabs PM100D) and maintained a constant value of 28 mW throughout all measurements.

Figure 4a,b shows the retrieved effective values for the imaginary (*k*_eff_) and real (*n*_eff_) components of the refractive index for our two-component IC-HT aerosol particles subjected to increasing exposures to 405 nm irradiation, with 1 min increments; it is important to note that during the short irradiation time of up to 360 s by the photolysis light source, the particle radius decreases by a negligible amount estimated to be ∼50 nm for the longest irradiation period. The *n*_eff_ remains nearly constant, with a mean value of 1.507 ± 0.004, but appears to decrease slightly with exposure time and tends to a value closer to 1.4906 ± 0.0012 reported for pure 1,2,6-hexanetriol at 405 nm.^48^ The values of *n*_eff_ and *k*_eff_ for the unbleached particles, at zero illumination time, were obtained in the absence of the photolyzing 405 nm laser, confirming that *k*_eff_ remains constant under nonirradiating conditions, despite the continual use of the 532 nm laser for phase function measurements and the 405 nm laser for the CRDS measurements.

**Figure 4:**
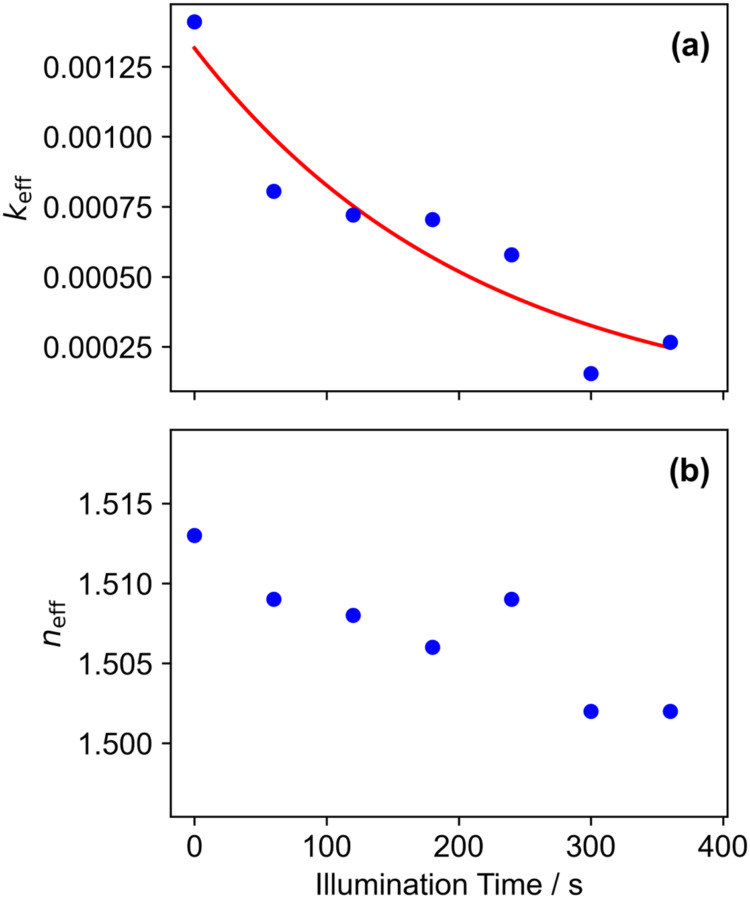
(a) Effect of 405 nm laser exposure time on the retrieved imaginary component of the refractive index values, *k*_eff_ (blue circles) for IC-HT droplets, with a fitted exponential function (red line). (b) The corresponding evolution in the real component of the droplet refractive index, *n*_eff_.

A value of *k*_IC_ = 0.00684 for pure IC is estimated from the experimentally measured value of *k*_eff_ for unbleached droplets. This value is calculated using the mass fraction mixing rule (eq 5), and assuming the mass ratio of the IC solute and HT in a droplet is identical to that in the bulk solution loaded into the droplet-on-demand dispenser. The calculation also assumes the same 45% conversion of IC to its hemiacetal form as used in our analysis of UV/vis spectra obtained for a bulk solution of IC in 1-butanol, because 1-butanol and 1,2,6-hexanetriol are both saturated aliphatic alcohols. This *k*_IC_ value compares favorably with the value *k*_IC_ = 0.0067 at 405 nm determined for the bulk solution of IC in 1-butanol (see above). The decrease in *k*_eff_ values with increasing exposure time demonstrates progressive photobleaching of IC. These retrieved *k*_eff_ values were fitted to a single exponential decay function of the form

6 $$k_{\mathrm{eff}}=k_{\mathrm{eff},0}\,\;\exp\left(-\mathrm{Bt}\right)$$

in which *k*_eff,0_ is the value of *k*_eff_ at *t* = 0 s and *B* characterizes the bleaching rate for *k*_eff_. This fitted exponential is shown in Figure 4a and has a decay constant of *B* = 0.0046 ± 0.0009 s^–1^.

### 3.2 Photochemical Modeling

The evolution of *k*_eff_ with photobleaching can be used to estimate the photobleaching quantum yield for IC in the droplet. We compare the evolving *k*_eff_ over the short period (of up to 360 s) of irradiation by the photolysis light source with a kinetic model that assumes the particle size is constant during irradiation; as discussed above, this is a reasonable approximation because we estimate that the particle radius decreases by up to ∼50 nm (i.e., a 3% decrease at most) during these irradiation periods. The photokinetics within small droplets are governed by the spatial and temporal distribution of light intensity inside the particle. For the droplets probed in our experiments, initial solute concentrations are expected to be consistent across all droplets given that they are all generated using a droplet-on-demand dispenser from stock solutions with identical compositions. Moreover, if molecular diffusion within the droplet is rapid, we can assume the solute distribution to be homogeneous throughout the droplet volume. With this latter assumption, we use a single-droplet photochemical model that is adapted from that described by Cremer et al.^32^

7 $$\frac{dN_{\mathrm{IC}}}{\mathrm{dt}}=-\frac{\Phi I_{0}}{\mathrm{hvV}}\sigma_{\mathrm{abs}}\left(N_{\mathrm{IC}}\right)$$

Here, *N*_IC_ is the number density of IC in a droplet (with units m^–3^), *t* is exposure time, Φ is the quantum yield, *I*_0_ is the intensity of the photolyzing light incident on a droplet, *h* is Planck’s constant, *v* is the frequency of the photolysis laser light, *V* is the droplet volume, and σ_abs_ is the absorption cross-section of the aerosol particle, which depends on the chemical composition of the droplet and therefore on *N*_IC_. Values for *k*_eff_ measured as a function of illumination time were reported in the previous section, and we need to adapt eq 7 to relate *N*_IC_ to *k*_eff_. Applying the mass fraction mixing rule to our IC-HT droplets, *k*_eff_ of the droplet is connected directly to the imaginary components of the refractive indices for the IC solute (in both its hemiacetal and aldehyde forms) by

8 $$k_{\mathrm{eff}}=w_{\mathrm{IC}}fk_{\mathrm{IC},A}+w_{\mathrm{IC}}\left(1-f\right)k_{\mathrm{IC},\mathrm{HA}}+\left(1-w_{\mathrm{IC}}\right)k_{\mathrm{HT}}$$

Here, *w*_IC_ is the mass fraction of the IC solute (for both its aldehyde and hemiacetal forms), *f* is the fraction of IC present in its aldehyde form with a corresponding imaginary refractive index of *k*_IC,A_, and *k*_IC,HA_ and *k*_HT_ are the imaginary refractive indices for the hemiacetal form of IC and 1,2,6-hexanetriol, respectively. In writing eq 8, we approximate that the molecular masses of IC are identical in both its forms. Both HT and the hemiacetal form of IC are nonabsorbing at the 405 nm wavelength (*k*_HT_ = 0, *k*_IC,HA_ = 0), and eq 8 simplifies to

9 $$k_{\mathrm{eff}}=w_{\mathrm{IC}}fk_{\mathrm{IC},A}$$

The mass fraction of IC is given by the ratio of the mass of IC within the droplet to the total droplet mass. The mass of IC in the droplet is *N*_IC_ *VM*_IC_/*N*_*A*_, with *V* the droplet volume, *M*_IC_ the molecular weight of IC (0.09609 kg mol^–1^), and *N*_A_ is the Avogadro constant. The total droplet mass is the product of the effective density of the droplet (ρ_eff_) and the droplet volume. Therefore, the mass fraction of IC in a droplet is

10 $$w_{\mathrm{IC}}=\frac{N_{\mathrm{IC}}M_{\mathrm{IC}}}{N_{A}\rho_{\mathrm{eff}}}$$

Combining eqs 9 and 10 and rearranging for *N*_IC_

11 $$N_{\mathrm{IC}}=\frac{N_{A}\rho_{\mathrm{eff}}}{M_{\mathrm{IC}}fk_{\mathrm{IC},A}}k_{\mathrm{eff}}$$

Assuming the effective density and volume of the droplet are independent of time during the 405 nm illumination (a good approximation for the short droplet irradiation times used in the experiments), we obtain

12 $$\frac{dk_{\mathrm{eff}}}{\mathrm{dt}}=-\frac{\Phi I_{0}M_{\mathrm{IC}}fk_{\mathrm{IC},A}}{\mathrm{hvV}N_{A}\rho_{\mathrm{eff}}}\sigma_{\mathrm{abs}}\left(k_{\mathrm{eff}}\right)$$

This nonlinear differential equation in *k*_eff_ may be solved analytically if σ_abs_(*k*_eff_) is a linear function of *k*_eff_, which we now explore.

Figure 5 shows the Lorenz-Mie theory calculations of the absorption cross-section with varying imaginary refractive index over the range 0.0000–0.0016 at a wavelength of 405 nm. The real part of the refractive index is set to 1.507 in all calculations, representing the mean value of the retrieved *n* for all droplets interrogated in this work. Three data sets for particle radii of 1300, 1400, and 1500 nm are displayed, with an additional data set showing the distribution corresponding to the mean initial particle radius of 1428 nm for the droplets interrogated. These calculations indicate that, over this *k*_eff_ range, it is reasonable to assume that σ_abs_ scales linearly with *k*_eff_, i.e., σ_abs_ = *gk*_eff_ with *g* values the gradients of the linear fits shown in Figure 5. Using this relationship between σ_abs_ and *k*_eff_ in eq 12, we write

13 $$\frac{dk_{\mathrm{eff}}}{\mathrm{dt}}=-\frac{\Phi I_{0}M_{\mathrm{IC}}fk_{\mathrm{IC},A}}{\mathrm{hvV}N_{A}\rho_{\mathrm{eff}}}gk_{\mathrm{eff}}$$

In its integrated form, eq 13 becomes

14 $$k_{\mathrm{eff}}=k_{\mathrm{eff},0}\exp\left[-\frac{\Phi I_{0}M_{\mathrm{IC}}fk_{\mathrm{IC},A}g}{\mathrm{hvV}N_{A}\rho_{\mathrm{eff}}}t\right]=k_{\mathrm{eff},0}\exp\left[-\frac{t}{\tau }\right]$$

in which

15 $$\tau =\frac{\mathrm{hvV}N_{A}\rho_{\mathrm{eff}}}{\Phi I_{0}M_{\mathrm{IC}}{\mathrm{fk}}_{\mathrm{IC},A}g}$$

with τ (in units of s) determined from our photobleaching experiments in Section 3.1. Therefore, the quantum yield is obtained via

16 $$\Phi =\frac{\mathrm{hvV}N_{A}\rho_{\mathrm{eff}}}{I_{0}M_{\mathrm{IC}}fk_{\mathrm{IC},A}g\tau }$$

in which *V* is calculated using $\frac{4}{3}\pi a^{3}$ , with the particle radius (*a*) estimated from the mean value of the initial particle size for our droplets determined from our recorded phase functions. ρ_eff_ may be estimated using the ideal mixing rule

17 $$\rho_{\mathrm{eff}}=\frac{1}{\frac{w_{\mathrm{IC}}}{\rho_{\mathrm{IC}}}+\frac{\left({1-w}_{\mathrm{IC}}\right)}{\rho_{\mathrm{HT}}}}$$

with ρ_IC_ the density of IC and ρ_HT_ the density of 1,2,6-hexanetriol. The value for *g* is taken from our Lorenz-Mie simulations of σ_abs_ vs *k*_eff_ for our mean initial particle radius of 1428 nm, for which *g* = (5.95 ± 0.36) × 10^–10^ m^2^. The value of *k*_IC,A_ is taken from our single aerosol particle analysis in Section 3.1. It corresponds to the IC solute in its aldehyde form in our 1,2,6-hexanetriol droplets in the absence of the photolysis light source and has a value of 0.00684. The incident light intensity *I*_0_ was calculated from the measured laser power (*P*_0_, 28.0 ± 0.2 mW) before our LEQ trap, in conjunction with the measured beam waist *w* (in units of m^2^), using the following formula

18 $$I_{0}=\frac{2P_{0}}{\pi w^{2}}$$

The Gaussian beam profile of the photolysis laser was measured at the position of the trapped particle using the following procedure. We trapped an ammonium sulfate droplet with a radius of ∼2 μm under constant humidity conditions (∼90% RH). Under these conditions, the trapped aerosol droplet maintained a constant size. The scattered light from the droplet was captured by the phase function imaging camera and, by using real time calculations in LabVIEW, an exposure feedback algorithm maintained the brightest pixel value within a narrow range of values. The trapping cell was mounted on a translation stage, the position of which was adjusted by control of a micrometre actuator (Thorlabs). As the position of the translation stage was adjusted to scan the location of the trapped particle across the profile of the photolysis beam, the exposure feedback was recorded. Figure 6 shows the difference in exposure time from its maximum value (averaged over a 10 s measurement period, with error bars representing the standard deviation over this 10 s window) with the position of the micrometre stage moved in 10 μm intervals. The red curve shows the best fit of a Gaussian distribution, which yielded a beam waist *w* = 137 ± 17 μm. This value is close to the expected beam waist of 154 μm based on geometric optics calculations that assume a 405 nm wavelength, a focal length of 60 cm for the lens focusing the photolyzing laser beam into the LEQ trapping cell, and a 1 mm beam diameter of the laser beam at the aperture of the focusing lens (with the specifications for the Toptica iBeam Smart stating a 1/*e* ^2^ beam diameter output of 1 mm). The discrepancy of ∼11% between our measured and geometric optics predicted values is within expected uncertainties from, for example, the beam diameter at the aperture of the focusing lens (the laser beam is expected to diverge slightly over the <1 m optical path length between the Toptica iBeam Smart laser head and the focusing lens). The determined beam waist of 137 ± 17 μm is used in combination with eqs 16–18 to calculate an effective photobleaching quantum yield for IC, under the approximation that the particle radius, effective particle density, and *g* parameter are constant throughout exposure to the photolysis light source. This effective quantum yield Φ for photobleaching of the IC-containing droplets is found to be (9.6 ± 3.0) × 10^–5^. The stated uncertainty corresponds to the propagation of uncertainties from all values used in the calculations of Φ, with Section S3 of the Supporting Information describing these uncertainties and providing an error sensitivity analysis. This analysis also accounts for micromotion of the droplet in the LEQ trap, which was characterized in prior work from our laboratory.^43^ The sensitivity analysis shows that the uncertainty in the quantum yield is dominated by uncertainties in the beam waist of the photolysis laser and in the best-fit time scale for photobleaching as determined from our exponential fit to the exposure time dependence of *k*_eff_ described in Section 3.1. The small value for Φ reflects the low propensity for IC to undergo irreversible photodegradation under 405 nm irradiation. Prior work has shown that IC exhibits moderate triplet state yields (0.61 ± 0.05 at pH = 7) and photochemical reactivity in aqueous environments, indicating the potential for photosensitized reactions but not necessarily rapid direct photobleaching.^20^ The present result suggests that, under our measurement conditions, photoexcited IC participates primarily in photochemical pathways that quench the excited state and recover the ground-state IC, rather than efficient unimolecular photolysis. In BrC aerosols, in particular at elevated concentrations of organic solutes, the triplet-state IC may instead undergo bimolecular reactions that accelerate its photochemical loss.

**Figure 5:**
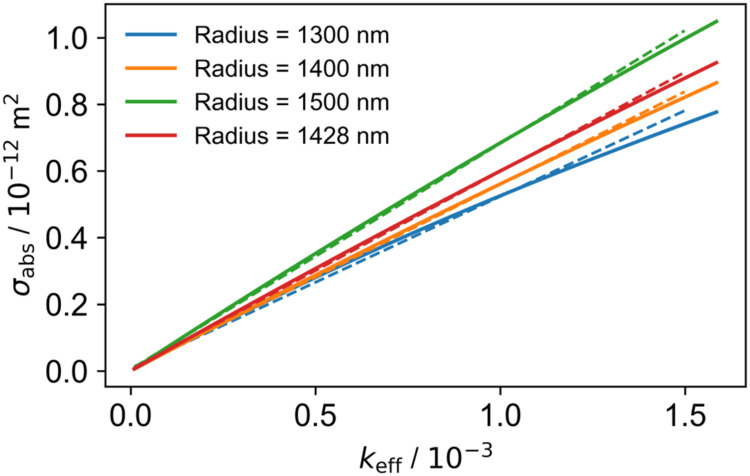
Lorenz-Mie calculations of absorption cross sections with a varying imaginary component of the refractive index at a wavelength of 405 nm. The real part of the complex refractive index is kept constant at 1.507. Data series show calculated distributions for four different values of particle radius, with the series for a particle radius of 1428 nm corresponding to the mean initial particle radius (i.e., when the droplets are first exposed to the photobleaching laser) for the droplets probed in this work. The dashed lines represent linear fits (constrained through the origin) to the calculated cross-section distributions over the *k* range 0–0.0015 that pertain to our measurements.

**Figure 6:**
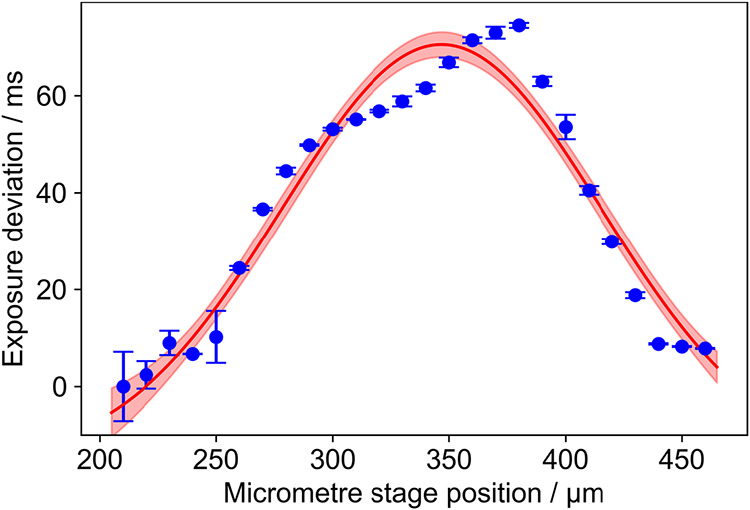
Exposure deviation measurements (blue points) with translation of a ∼2 μm radius ammonium sulfate particle in 10 μm intervals through the profile of the photolyzing laser beam. The red curve represents the best fit of a Gaussian beam profile, with the 1σ uncertainty shown by the red shaded area.

To compare this effective photobleaching quantum yield measured in a droplet with the corresponding behavior in the bulk liquid phase, we conducted solution-phase photobleaching experiments using UV/vis absorption spectroscopy to analyze a degassed 3 mL sample – corresponding to 1-butanol containing 0.02% IC by mass fraction – in a sealed cuvette after continuous exposure to 405 nm radiation from the same photolysis laser source used in our single aerosol particle experiments (see SI Section S2 for experimental details and uncertainty analysis). These bulk experiments were performed in 1-butanol that, like 1,2,6-hexanetriol, is a saturated alcohol species. 1-butanol was chosen because of the practical difficulties of mixing IC in a pure 1,2,6-hexanetriol solvent owing to its high viscosity. After accounting for the small amount of evaporation of the solvent over the extended reaction period (>100 h), the bulk solution-phase quantum yield is estimated to be (2.1 ± 0.1) × 10^–6^, under the assumption that no significant photoproduct accumulation contributes to the observed absorbance changes and the same 45% conversion of the IC to its hemiacetal form. An upper-limit estimate of (2.2 ± 0.6) × 10^–5^ is obtained when the calculation is based on the absorbance decrease at 405 nm; in this case, the potential loss of aggregate-state IC molecules may contribute to the apparent signal change. Regardless of the estimation approach, the quantum yield measured in the bulk solution remains substantially lower than the effective quantum yield determined in the single aerosol particle experiments. We do not attribute this discrepancy to the presence of dissolved O_2_ in the droplet for two reasons. First, the single-particle experiments were conducted under a continuously N_2_-purged atmosphere, thereby minimizing the potential influence of molecular oxygen. Second, control measurements performed on bulk solutions without prior degassing yielded a quantum yield of (2.1 ± 0.7) × 10^–6^, which showed no statistically significant difference from those obtained for degassed bulk samples. In both cases, the bulk-phase values were consistently lower than those measured in the aerosol experiments. These observations indicate that dissolved oxygen is unlikely to account for the enhanced photochemical reactivity observed in the aerosol phase. Effects of aerosol specific phenomena in accelerating photodegradation, such as the role that interfacial phenomena may play at the particle interface that might open alternative photochemical pathways, cannot be eliminated. In addition, the confined geometry and rapid solvent evaporation associated with aerosol particles may promote aggregation of IC, potentially altering its photochemical behavior relative to the bulk solution.

## 4 Conclusions

We have presented the first direct measurements of photobleaching kinetics for imidazole-2-carboxaldehyde, a key imine brown carbon species, in single organic aerosol droplets using single particle cavity ring-down spectroscopy (SP-CRDS). By accurately retrieving the imaginary component of the refractive index at a wavelength of 405 nm while irradiating the droplet with a 405 nm diode laser beam over a range of controlled exposure times, we demonstrate significant reductions in light absorptivity due to photochemical aging. To interpret these observations, we developed a photokinetic model which accounts for particle size and spatial variation in the incident light intensity. Additionally, we proposed a novel experimental approach to measure the laser beam profile inside an electrodynamic trap, enabling an accurate estimation of the irradiance at the position of our interrogated particles, from which we determine the effective photobleaching quantum yield (within the framework of our developed kinetic model) of the imidazole-2-carboxaldehyde in the droplet. The methodology and modeling framework developed here are generalizable to a wide class of BrC species and other light-absorbing aerosol systems, opening opportunities to explore the effects of unique aerosol properties on their photochemistry. This study advances the understanding of BrC aging mechanisms and offers valuable data to constrain aerosol-climate interactions in atmospheric models.

## Supplementary Material



## Notes

Particle complex refractive index retrieval; bulk solution quantum yield measurement; sensitivity of effective photobleaching quantum yield determined from in-aerosol measurements; and assignment of imidazole-2-carboxaldehyde as the absorber of the 405 nm light from the photobleaching laser (PDF)
