# Supplementary material for: Photobleaching of Imidazole Brown Carbon in Single Levitated Aerosol Particles
Source: J Phys Chem A. 2026 Jun 1;130(23):4401–10. doi: 10.1021/acs.jpca.6c01327 (PMC13267089; doi:10.1021/acs.jpca.6c01327)
Supplement: Supplementary file 1 [file jp6c01327_si_001.pdf]

Supporting Information

Photobleaching of Imidazole Brown Carbon in Single Levitated Aerosol Particles

Xu Zhang,[1] Padraig E. Meehan,[3] Jamie W. Knight,[1,2] Andrew J. Orr-Ewing,[1*]
and Michael I. Cotterell[3]*

[1]School of Chemistry, University of Bristol, Bristol, BS8 1TS, UK

[2]College for Engineering, Mathematics and Physical Sciences, University of Exeter, Exeter, EX4 4QF, UK,

[3]Department of Chemistry, University of Oxford, Oxford, OX1 3QZ, UK

*Correspondence to:
a.orr-ewing@bristol.ac.uk
michael.cotterell@chem.ox.ac.uk

S1 Particle Complex Refractive Index Retrieval

This section provides additional detail on the optimization strategies used to retrieve the complex refractive indices of single levitated droplets from size-dependent extinction cross-section measurements.

As discussed in Section 2.2 of the main text, the numerical behaviour of the established merit function exhibited limited sensitivity to the magnitude of the imaginary refractive index, k, which led to reduced robustness in retrieving k for weakly absorbing and non-absorbing particles. For the unbleached droplets and those in the early stages of photobleaching (with $k >$ 0.0008), we had applied our previously established simultaneous optimization scheme for retrieving the complex refractive index. Examples of the corresponding merit contour plots for this regime are shown in Figure S1. In all cases, the allowed range of k was constrained by the interference structure and resonance-peak amplitudes present in the σ_{ext} curves. This physically motivated constraint was introduced to compensate the limitation of the optimization algorithm. Well-defined minima were observed in n, with the minimum χ values lying within the expected order of magnitude ($\sim 10^{-26}$ m^4), consistent with the experimental uncertainty in the extinction cross-section measurements.

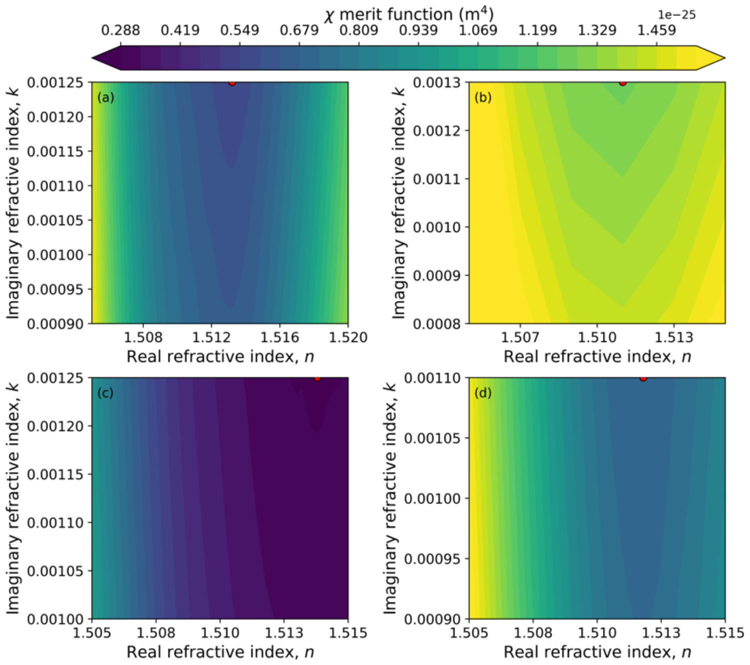

Figure S1. Contour plots obtained using the established simultaneous optimization scheme for IC–HT droplets with k>0.0008. Panels (a)–(c) show three independent unbleached droplets, while panel (d) corresponds to a droplet after 1 min of photobleaching. The red markers indicate the global minima of the χ-merit function.

While this optimization scheme provides well-constrained values of the real refractive index n, the retrieval of the imaginary refractive index is inherently limited. As shown in Figure S1, the minima of the χ-merit function consistently occur at or near the boundaries of the explored k range, indicating that the simultaneous optimization does not yield a well-defined optimum value for k under these conditions. This behaviour reflects the reduced sensitivity of σ_{ext} to absorption when k becomes sufficiently small, such that variations in k no longer produce a distinct minimum in χ within the accessible parameter space. Consequently, although the allowed k range can be constrained to remain physically reasonable, the simultaneous optimization scheme is unable to provide a robust or unique determination of k in this weakly absorbing regime. For IC–HT droplets with k<0.0008, the simultaneous optimization of n, k, w, and r_m becomes increasingly ill-conditioned, as σ_{ext} exhibits only weak dependence on the imaginary refractive index. In this regime, variations in k primarily affect the resonance amplitudes, while the interference structure and peak positions remain dominated by the real refractive index n.

To address this limitation, a two-stage optimization approach was employed. In the first stage, a three-parameter grid search was performed over n, w, and r_m, while k was fixed at a small constant value. This simplification exploited the fact that, for sufficiently small absorption, the size-dependent interference structure in σ_{ext} provided robust constraints on n, w, and r_m within our experiment size range. In the second stage, the imaginary refractive index was refined independently using an amplitude-based merit function that minimized the difference between the experimentally measured and Lorenz–Mie-predicted maximum extinction values. Representative k-merit functions obtained during this second optimization stage are shown in Figure S2. Compared with a direct four-parameter grid search using comparable grid densities, the staged optimization yielded systematically lower and more physically reasonable values of k. In addition, reducing the dimensionality of the initial grid search substantially

decreased the total number of required grid points, enabling a finer scan of the imaginary refractive index and, consequently, a more precise determination of k in the weakly absorbing and effectively non-absorbing regime.

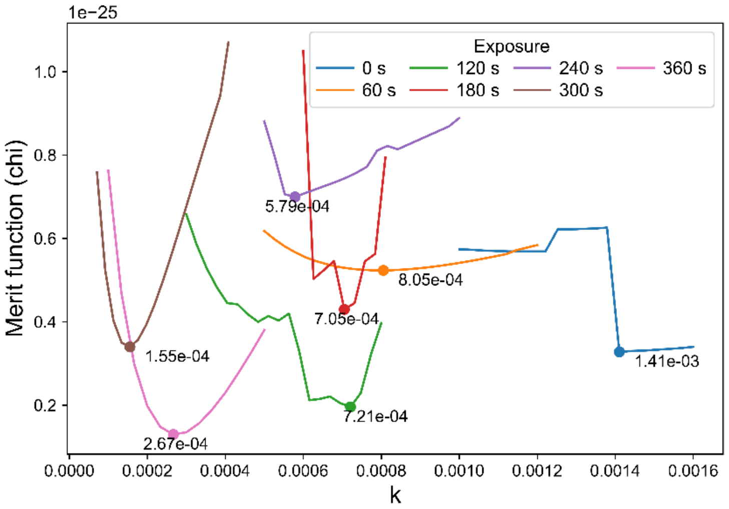

Figure S2. Imaginary refractive index (k) merit functions obtained from the staged optimization procedure for IC–HT droplets. In all cases, a single, well-defined minimum is observed, enabling a stable determination of the imaginary refractive index.

S2 Bulk Solution Quantum Yield Measurement

This section describes the experimental framework employed to determine the photobleaching quantum yield of IC in bulk solution, together with a systematic evaluation of the associated uncertainties.

Prior to cuvette loading, a 3 mL aliquot of IC dissolved in 1-butanol (mass fraction 0.02%) was degassed by repeated freeze–pump–thaw cycles. In this procedure, the solution was frozen, evacuated under vacuum, and thawed repeatedly to remove dissolved gases from the solvent. The solution was then transferred into a quartz cuvette with an optical path length of 1 cm, a micro stirrer bar was added, and the cuvette was sealed with a bung and Parafilm. The cuvette was placed on a magnetic stirrer to ensure homogeneous mixing throughout the photolysis experiment. A continuous-wave 405-nm laser beam was directed through the cuvette to initiate the photochemical reaction, while the incident and transmitted power were continuously monitored using a calibrated power meter. Under these conditions, the photon flux absorbed by the solution was quantified by monitoring the change in laser power before and after the cuvette was filled:

$$P_{\mathrm{abs}} = P_{\mathrm{inc}} - P_{\mathrm{trans}} - P_{\mathrm{refl}} \tag{S1}$$

where P_{abs} is the power absorbed by the solution. P_{inc}, P_{trans} and P_{refl} are the power of the incident light beam, the transmitted power and the reflected power, respectively. In this analysis, energy losses due to the scattering at the interface of the solution surface and cuvette were considered negligible. The energy associated with each photon, E_{ph}, was determined from its wavelength using:

$$E_{\mathrm{ph}} = \frac{hc}{\lambda} \tag{S2}$$

where h is Planck's constant, c is the speed of light, and λ is the wavelength of the incident radiation. The absorbed photon count was then calculated according to the following expression:

$$N_{\mathrm{abs}} = \frac{P_{\mathrm{abs}} \times t}{E_{\mathrm{ph}}} \qquad (S3)$$

in which N_{abs} is the number of photons absorbed by the solution, and t is the exposure time. The temporal evolution of the IC concentration was determined using the Beer–Lambert law. Following preliminary experiments to estimate the characteristic timescale of photobleaching, the cuvette was periodically removed from the irradiation setup and its absorption spectrum was recorded using a UV/Vis spectrometer. We assume that photobleaching products do not contribute to absorption at the IC maximum (282 nm). This assumption is supported by the invariant spectral shape over time (Figure S3). Consequently, the decrease in absorbance at 282 nm directly reflects the loss of IC molecules. Figure S3 depicts the absorbance change with time.

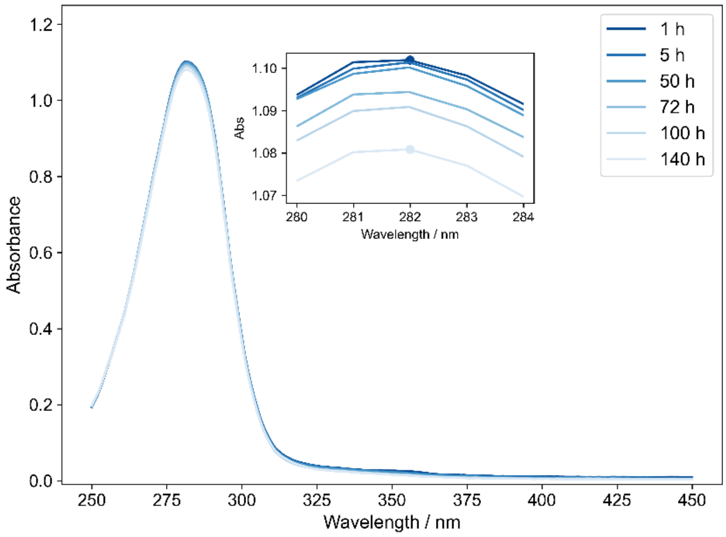

Figure S3. Time-dependent absorbance changes of the IC–1-butanol solution subjected to irradiation by a cw 405-nm laser. The inset expanded plot highlights the decrease of the 282 nm absorption band during photobleaching over a 140-hour period of continuous irradiation.

A decrease of 1.9% in the absorbance at 282 nm was observed, corresponding directly to the change in IC concentration. Over the 140-hour irradiation period, solvent evaporation, enhanced by continuous laser exposure and magnetic stirring, resulted in a small but measurable decrease in solvent volume. This evaporation leads to an effective increase in IC concentration, partially compensating for IC loss due to photobleaching.

The principal sources of uncertainty in this experiment include solvent evaporation, reflection losses from the cuvette and solution interfaces, and variations in the absorbed laser power arising from changes in solute concentration during photobleaching. Given the dominant impact of solvent evaporation on the observed concentration changes, only this contribution is considered in the present uncertainty analysis. The decrease in solvent volume is measured to be 6.7 ± 1.7%, and this range is used to assess the resulting uncertainty in the calculated IC consumption. Accordingly, the photobleaching quantum yield and its uncertainty can be evaluated using the following definition:

$$\Phi(\lambda)=\frac{moles\ of\ product\ formed\ (or\ reactant\ consumed)}{moles\ of\ photons\ absorbed\ at\ \lambda} \quad (S4)$$

From this analysis, the photobleaching quantum yield for IC in bulk 1-butanol solution at 405 nm was determined to be $(2.1 \pm 0.6) \times 10^{-6}$. This calculated quantum yield also accounts for the conversion of 45% of the IC molecules to their hemiacetal form.

To examine whether dissolved oxygen influences the bulk-solution photobleaching quantum yield, an additional control experiment was performed using an IC–1-butanol solution prepared without prior degassing. The experimental procedure and analysis were otherwise identical to those described above. The temporal evolution of the absorbance spectrum for the non-degassed solution is shown in Figure S4. As for the degassed sample, the spectral shape remains unchanged during irradiation, and the decrease in absorbance at 282 nm was used to quantify IC consumption. Small differences in spectral shape are observed near the edges of the absorption band for the 75 and 100 h data sets. These variations are most likely associated with slow changes in the spectral baseline over the course of the week-long measurement, arising from minor instrumental drift and background fluctuations rather than from chemical changes in the IC absorption profile. Importantly, the position and shape of the main absorption band remain unchanged. It is also noted that the absorbance at the band maximum (282 nm) is not strictly monotonic with irradiation time and, in some cases, slightly exceeds the initial value. This behaviour is attributed to solvent evaporation from the sealed cuvette, which leads to an effective increase in IC concentration that partially compensates for photochemical loss. Because the evaporation rate is not constant and is largest during the early stages of the experiment, small non-monotonic variations in the measured absorbance are expected. These effects are accounted for in the uncertainty analysis described above and do not affect the validity of using the absorbance change at 282 nm to quantify IC consumption.

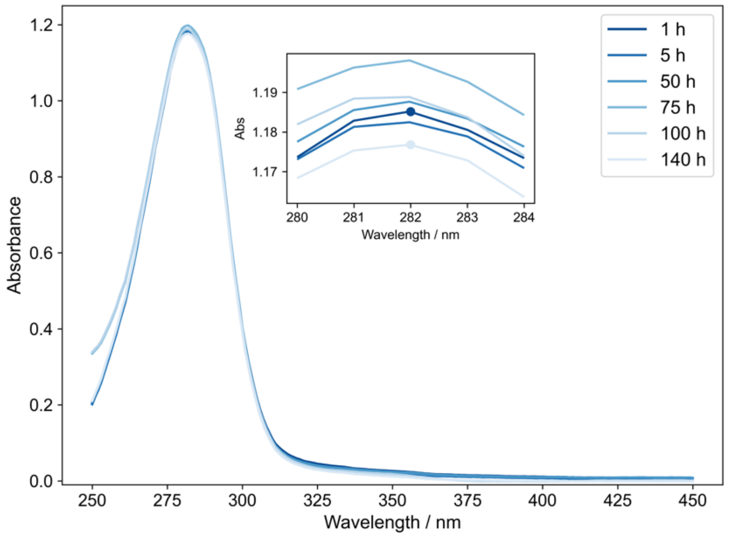

Figure S4. Time-dependent UV/Vis absorption spectra of an IC–1-butanol solution prepared without prior degassing and subjected to continuous irradiation at 405 nm. The inset expanded plot highlights the decrease of the 282 nm absorption band during photobleaching over a 140-hour period of continuous irradiation.

The photobleaching quantum yield derived from the absorbance change at 282 nm for the non-degassed solution was $(2.1 \pm 0.7) \times 10^{-6}$, which is statistically indistinguishable from that

obtained for the degassed bulk solution. This result indicates that the presence of dissolved oxygen does not significantly affect the bulk-solution photobleaching quantum yield under the conditions of this study.

To further evaluate the reliability of the bulk-solution photobleaching quantum yield, wavelength-resolved quantum yields were derived from full UV/Vis absorption spectra recorded at selected irradiation times (50, 72, 100, and 140 h). The resulting quantum yield spectra are shown in Figure S5. Across the wavelength range investigated (250–450 nm), the quantum yields obtained at different irradiation times exhibit consistent magnitudes and similar spectral trends, indicating that the photochemical efficiency of IC in bulk 1-butanol does not vary systematically as photobleaching progresses.

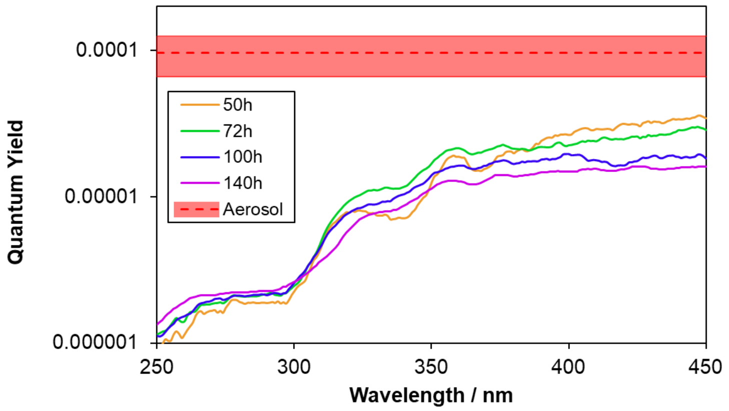

Figure S5. Wavelength-resolved photobleaching quantum yields of IC in bulk 1-butanol solution derived from full UV/Vis absorption spectra recorded after 50, 72, 100, and 140 h of continuous irradiation at 405 nm. The shaded red region indicates the range of quantum yields obtained for single aerosol particles, shown for comparison. The similarity of the spectra at different irradiation times indicates that the bulk-solution quantum yield remains stable throughout the photobleaching experiment.

An overall increase in the apparent quantum yield is observed toward longer wavelengths in the range 310–450 nm. In particular, quantum yields estimated from absorbance changes near 405 nm approach the values measured for single aerosol particles. However, in this spectral region the absorbance of IC is very small and, in some cases, approaches the detection limit of the 8-bit UV/Vis spectrometer. Consequently, the derived quantum yields at longer wavelengths are subject to substantially larger uncertainty. Despite this wavelength-dependent variability, over the strong IC absorption band (250–310 nm), the wavelength-resolved quantum yields are nearly identical and fall within the same order of magnitude as the average value derived from the absorbance decrease at 282 nm. This supports the validity of using the single-wavelength analysis at the IC absorption maximum to determine the representative bulk-solution quantum yield reported in this study.

S3 Sensitivity of effective photobleaching quantum yield determined from in-aerosol measurements

We performed a sensitivity analysis to understand how our calculated value of the effective photobleaching quantum yield for our IC-1,2,6-hexanetriol aerosol droplets depended on the uncertainties in the values used in this calculation. The uncertainties in the parameters used in

this calculation are summarized in Table S1 below. In our quantum yield calculation, the particle radius at the beginning of the measurement, during which the droplet is exposed to the photolysis light source, is assumed to be constant and approximated as the mean initial radius across all the aerosol droplets measured. This mean initial radius was 1428 µm with a standard deviation of 56 nm. The density of IC is needed in the ideal mixing rule calculation of the effective droplet density. The density of IC is stated by the manufacturer as (1322 ± 0.060) g cm^{-3}, but this is a predicted value and the predictive method used is not available. The provided uncertainty of ~5% is small and we expect that this uncertainty is larger; we therefore take the uncertainty in the density of IC to be 10%. The photolysis laser power P_0 was (28.0 ± 0.2) mW, with the uncertainty corresponding to the standard error in the Thorlabs PM100D photodiode measurement of power. The determination of the beam waist as (137 ± 17) µm is described in the main text. The value of $k_{IC,A}$ is estimated to have an uncertainty of ~10%. The g value, describing the gradient of the absorption cross-section with the effective imaginary refractive index of the droplet, was determined in the main text to be (5.95 ± 0.36) × 10^{-10} m^2. The photobleaching timescale τ was calculated from our fit of the equation $k_{\text{eff}} = k_{\text{eff,0}} \exp(-t/\tau)$ to the exposure time dependence of our retrieved imaginary components of the refractive index, k_{eff}, as described in Section 3.1 of the main text; the best-fit τ was (217 ± 44) s, with the error corresponding to the one-sigma standard error in the best-fit parameter obtained from the covariance matrix of the fit.

**Parameter**	**Uncertainty in parameter**	**Corresponding percentage uncertainty in effective photobleaching quantum yield**
Mean Droplet Radius	56 nm	12.3%
Density of IC	10%	3.2%
Photolysis laser power output	0.2 mW	0.7%
Beam waist	17 µm	26.4%
$k_{IC,A}$	10%	11.1%
g parameter	3.6 × 10^{-11} m^2	5.7%
Best fit photobleaching timescale, τ	44 s	25.6%

Table S1. Summary of the parameters used in our effective photobleaching quantum yield calculation for our 1,2,6-hexanetriol droplets, their uncertainties, and the corresponding error introduced to the calculated photobleaching quantum yield expressed as a percentage in the mean value.

The results of this sensitivity analysis reveal that the uncertainty in the quantum yield is dominated by those in the beam waist of the photolysis laser and in the best-fit photobleaching timescale. We highlight that micromotion of particles levitated within an electrodynamic balance can make their spectroscopic interrogation challenging and could perturb the effective exposure of the particle to the photolyzing light source. We quantified the amplitude of this micromotion in a previous publication,[4] with this amplitude determined to be 68 µm. This micromotion likely contributes to the 'double-humped' structure in Figure 6 of the main text. Because we used a levitated aerosol particle to characterize the effective beam waist of the photolysis laser at the position of the trapped particle, the effect of micromotion on this effective beam waist will – to a degree - be accounted in our estimation of the photobleaching

quantum yield and its uncertainty. However, if we assume that micromotion imparted an even larger uncertainty on the effective beam waist than that already considered, by doubling the uncertainty in the beam waist from the value considered in Table S1, the resulting *uncertainty* in the effective photobleaching quantum yield increases by ~50%; this increase in uncertainty does not impact the conclusions of our work.

S4 Assignment of imidazole-2-carboxaldehyde as the absorber of the 405-nm light from the photobleaching laser

Figure 2 of the main manuscript contains absorption spectra of aqueous and 1-butanol solutions of IC, with the strongest absorption bands peaking at wavelengths below 300 nm. The inset to Figure 2(a) shows that absorption by the prepared solutions extends to wavelengths above 400 nm. Evidence from high-level (MRCI/cc-pVTZ//B3LYP/6-31G(d)) electronic structure calculations by Martins-Costa *et al.* supports the assignment of this longer-wavelength absorption to a weak $n\rightarrow\pi^*$ electronic transition associated with the carbonyl group of IC.[1] These authors calculated vertical excitation energies (from the frozen ground-state geometries) for the first two absorption bands of IC at 4.09 eV (λ = 303 nm, $n\rightarrow\pi^*$) and 4.73 eV (λ = 262 nm, $\pi\rightarrow\pi^*$). Although the first band is calculated to have zero oscillator strength, the $n\rightarrow\pi^*$ transitions become weakly electric-dipole allowed through zero-point vibrational motions of the ground-state molecules, solvent-solute interactions that break the planar symmetry, and vibronic coupling in the S_1 state. Incorporating such effects, and spectral shift to longer wavelength caused by water solvation, Martins-Costa et al. present a computed (QM/MM combined with MRCI/cc-pVTZ) absorption spectrum for aqueous IC in Figure 8 of their paper. This absorption spectrum extends to wavelengths beyond 400 nm, albeit with absorption cross sections that are a factor of more than 100 less than that of the band maximum near 287 nm. The ratio of the calculated absorption cross sections at 400 nm and at the band maximum is ~ 0.002. In our experimental spectra, the corresponding ratio is 0.005, in satisfactory agreement with the computational prediction and consistent with absorption by IC in the long-wavelength wing of the band.

We used our absorbance spectra for IC in aqueous solution, presented in Figure 2 of the main text, to calculate the absorption cross-section for the purposes of comparison with the values predicted by Martins-Costa et al. Our calculated absorption cross-section is 2.0×10^{-19} cm^2 $molecule^{-1}$, which is in reasonable agreement with the value of 1.09×10^{-19} cm^2 $molecule^{-1}$ reported by Martins-Costa *et al*.

The absorption spectra of carboxylic acids like nonanoic acid have been shown to be vulnerable to impurities, requiring extensive purification to ensure reliable absorption spectra of the pure samples in solution.[2] For the carboxylic acids, impurity absorptions at wavelengths above 250 nm arise from small amounts of contaminant organic carbonyl compounds. Interfering absorptions by impurity organic compounds are less likely to arise at wavelengths around 400 nm relevant to the current study of IC because fewer organic chromophores are expected at such long wavelengths. Nevertheless, the purity of the IC sample (manufacturer specified to be > 97 %) was checked by ^1H and ^{13}C NMR spectroscopy in DMSO-d_6. The spectra show the expected signals for the aldehyde and the aromatic ring,[3] with only minor additional resonances attributable to trace impurities and residual solvent. Overall, the NMR data are consistent with the nominal purity. The corresponding spectra are shown in Figure S6.

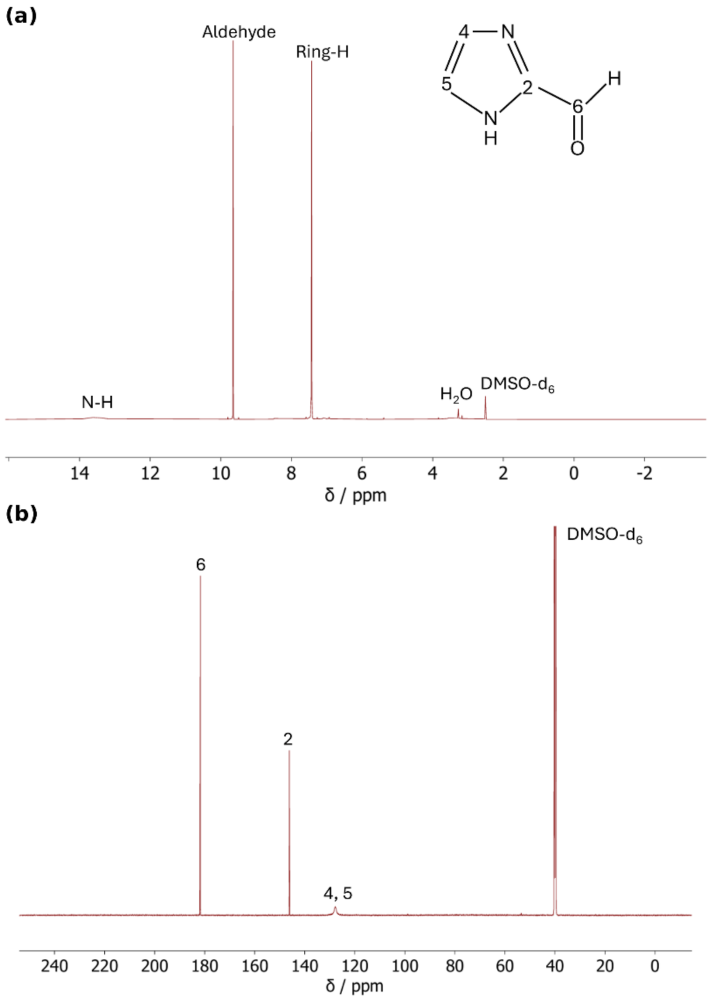

Figure S6. ^{1}H (a) and ^{13}C (b) NMR spectra of IC recorded in DMSO-d$_6$.

Our UV/Vis spectra of IC in bulk solutions show a weak feature at wavelengths from ~380 - 440 nm. This band may be a weak shoulder of the IC n $\rightarrow$ π^* transition or a signature of J-aggregates of IC forming at low concentrations.

References

(1) Martins-Costa, M. T. C.; Anglada, J. M.; Francisco, J. S.; Ruiz-Lopez, M. F. Photosensitization mechanisms at the air-water interface of aqueous aerosols. *Chem Sci* **2022**, *13* (9), 2624-2631. DOI: 10.1039/d1sc06866k From NLM PubMed-not-MEDLINE.

(2) Saito, S.; Numadate, N.; Teraoka, H.; Enami, S.; Kobayashi, H.; Hama, T. Impurity contribution to ultraviolet absorption of saturated fatty acids. *Sci Adv* **2023**, *9* (38), eadj6438. DOI: 10.1126/sciadv.adj6438 From NLM PubMed-not-MEDLINE.

(3) Lázaro Martínez, J. M.; Romasanta, P. N.; Chattah, A. K.; Buldain, G. Y. NMR Characterization of Hydrate and Aldehyde Forms of Imidazole-2-carboxaldehyde and Derivatives. *The Journal of Organic Chemistry* **2010**, *75* (10), 3208-3213. DOI: 10.1021/jo902588s.

(4) Knight, J.W. et al., Evaluating the Accuracy of Absorbing Aerosol Optical Properties Measured using Single Particle Cavity Ring-Down Spectroscopy", *Aerosol Science and Technology*, 2023, **57**(5): 406-424
